# Evaluation of liver parenchyma and perfusion using dynamic contrast-enhanced computed tomography and contrast-enhanced ultrasonography in captive green iguanas (*Iguana iguana*) under general anesthesia

**DOI:** 10.1186/1746-6148-10-112

**Published:** 2014-05-13

**Authors:** Giordano Nardini, Nicola Di Girolamo, Stefania Leopardi, Irene Paganelli, Anna Zaghini, Francesco C Origgi, Massimo Vignoli

**Affiliations:** 1 Veterinary Clinic Modena Sud, Spilamberto, MO 41057, Italy; 2 Clinica per Animali Esotici, CVS, Rome 00137, Italy; 3 Department of Veterinary Medical Sciences, University of Bologna, Ozzano dell’Emilia, BO 40064, Italy; 4 Center for Fish and Wildlife Health (FIWI), College of Veterinary Medicine, University of Bern, Vetsuisse Faculty, Bern, CH 3012, Switzerland; 5 PetCare Veterinary Association, Marzabotto, BO 40043, Italy

## Abstract

**Background:**

Contrast-enhanced diagnostic imaging techniques are considered useful in veterinary and human medicine to evaluate liver perfusion and focal hepatic lesions. Although hepatic diseases are a common occurrence in reptile medicine, there is no reference to the use of contrast-enhanced ultrasound (CEUS) and contrast-enhanced computed tomography (CECT) to evaluate the liver in lizards. Therefore, the aim of this study was to evaluate the pattern of change in echogenicity and attenuation of the liver in green iguanas (*Iguana iguana*) after administration of specific contrast media.

**Results:**

An increase in liver echogenicity and density was evident during CEUS and CECT, respectively. In CEUS, the mean ± SD (median; range) peak enhancement was 19.9% ± 7.5 (18.3; 11.7-34.6). Time to peak enhancement was 134.0 ± 125.1 (68.4; 59.6-364.5) seconds. During CECT, first visualization of the contrast medium was at 3.6 ± 0.5 (4; 3-4) seconds in the aorta, 10.7 ± 2.2 (10.5; 7-14) seconds in the hepatic arteries, and 15 ± 4.5 (14.5; 10-24) seconds in the liver parenchyma. Time to peak was 14.1 ± 3.4 (13; 11-21) and 31 ± 9.6 (29; 23-45) seconds in the aorta and the liver parenchyma, respectively.

**Conclusion:**

CEUS and dynamic CECT are practical means to determine liver hemodynamics in green iguanas. Distribution of contrast medium in iguana differed from mammals. Specific reference ranges of hepatic perfusion for diagnostic evaluation of the liver in iguanas are necessary since the use of mammalian references may lead the clinician to formulate incorrect diagnostic suspicions.

## Background

Although in reptiles hepatic diseases are traditionally suspected to be secondary to husbandry mismanagement, hepatic diseases caused by parasitic infestations [1], viral [2-4] and bacterial [5,6] infections, and neoplastic proliferations [7,8] are sporadically reported in the literature.

Even though hepatic diseases are quite common in reptiles they are hardly diagnosed ante mortem due to the ambiguous clinical signs [2,4,5]. Furthermore, the function of the reptilian liver is markedly influenced by age, sex, physiological condition, temperature, and other environmental conditions [9]. Changes in the organ’s size, color, and texture, as well as alterations of biochemical parameters [10] may therefore be alternatively considered either normal or related to hepatic diseases. For example, in chelonians there are physiological fluctuations of liver enzymes that depend on the seasons and on the gender [11,12]. Therefore, the diagnosis of liver pathological conditions is challenging and evaluation of the liver through multiple approaches is often necessary.

In green iguanas (*Iguana iguana*) an effective endoscopic technique for collection of liver biopsy specimens has been described [13]. Endoscopic biopsy is a powerful technique, especially when dealing with multifocal/diffuse diseases. Unfortunately, there are no data in the current literature describing the sensitivity of liver endoscopic biopsy when dealing with focal hepatic disease. Although to achieve a definitive diagnosis tissue biopsy may be required, a preliminary characterization of liver parenchyma and perfusion by use of non-invasive imaging techniques may be useful to evaluate the presence of lesions, to localize them and to determine the extent of their distribution.

In companion animal medicine, several techniques allow morphological evaluations of the liver. Among them, ultrasonography has been widely employed, while computed tomography (CT) and contrast-enhanced computed tomography (CECT) are being increasingly utilized [14]. Although ultrasonography provides details on the morphology and the vascularization of the liver, contrast-enhanced ultrasound (CEUS), based on the injection of specific contrast agents, allows the investigation of the perfusion of tissues [15]. When a tissue is perfused with an ultrasound contrast agent, analysis of grayscale images collected over an appropriate duration of time permits creation of time–intensity curves for a chosen region of interest. Mathematical analysis of these time–intensity curves yields quantitative hemodynamic indices relating to blood flow in either tissue volumes or within individual vessels [15]. Perfusion parameters are of interest since changes in vascularity and blood flow secondary to pathology generate alterations in the time–intensity curve [16]. Furthermore, contrast-enhanced ultrasound allows a more complete characterization of focal lesions [17].

In canine patients contrast-enhanced ultrasonography appears to improve the characterization of focal and multifocal hepatic lesions [18] and in humans CEUS detected significantly more focal liver lesions than unenhanced ultrasonography [19]. Apart for imaging of the liver [17,18] CEUS has also been demonstrated to be a useful for imaging other organs including canine spleen [17,20], lymph nodes [21], prostate [22,23] kidneys [24,25], and adrenals [26].

Computed tomography may also provide useful clinical information when used to evaluate perfusion of hepatic parenchyma and of focal hepatic lesions [27,28]. Although in reptiles CT has been historically used to describe the normal anatomy [29-33], more recently, probably due to the easier access to CT scanners, it has been employed for clinical evaluation of anatomical changes [34,35]. Therefore, description of reference ranges for liver hemodynamics of healthy individuals may be useful to permit assessment of the liver in clinical settings.

Although both CECT and CEUS could be powerful diagnostic tools to evaluate liver morphology and perfusion, to the best of the authors’ knowledge, no studies have so far evaluated their use in reptiles. Therefore, the aim of this study was to evaluate architecture and perfusion of the liver in green iguanas (*Iguana iguana*) by use of CECT and CEUS, to serve as a reference for future clinical studies. The specific hypothesis was that distribution of the contrast media after intravenous injection in a peripheral vein would result in a rise in echogenicity and attenuation of the liver, and therefore CECT and CEUS would be useful tools to evaluate liver perfusion.

## Results

### Population summary

Of eleven iguanas presented in the study period, eight (7 males, 1 female) met the criteria for inclusion in the study. Two iguanas were excluded for clinical abnormalities, and one iguana was excluded due to abnormal values on serum biochemistry. Mean age of the included iguana was 9 years (range 2-18 yr), and mean body weight was 1.9 kg (range 1.1-2.8 kg). All the iguanas recovered uneventfully from anesthesia and no notable complications were associated with the procedure. Histological examination did not reveal the presence of significant tissue changes except for a mild to moderate degree of hepatic lipidosis detected in all the liver samples observed.

### CEUS

**Figure 1:**
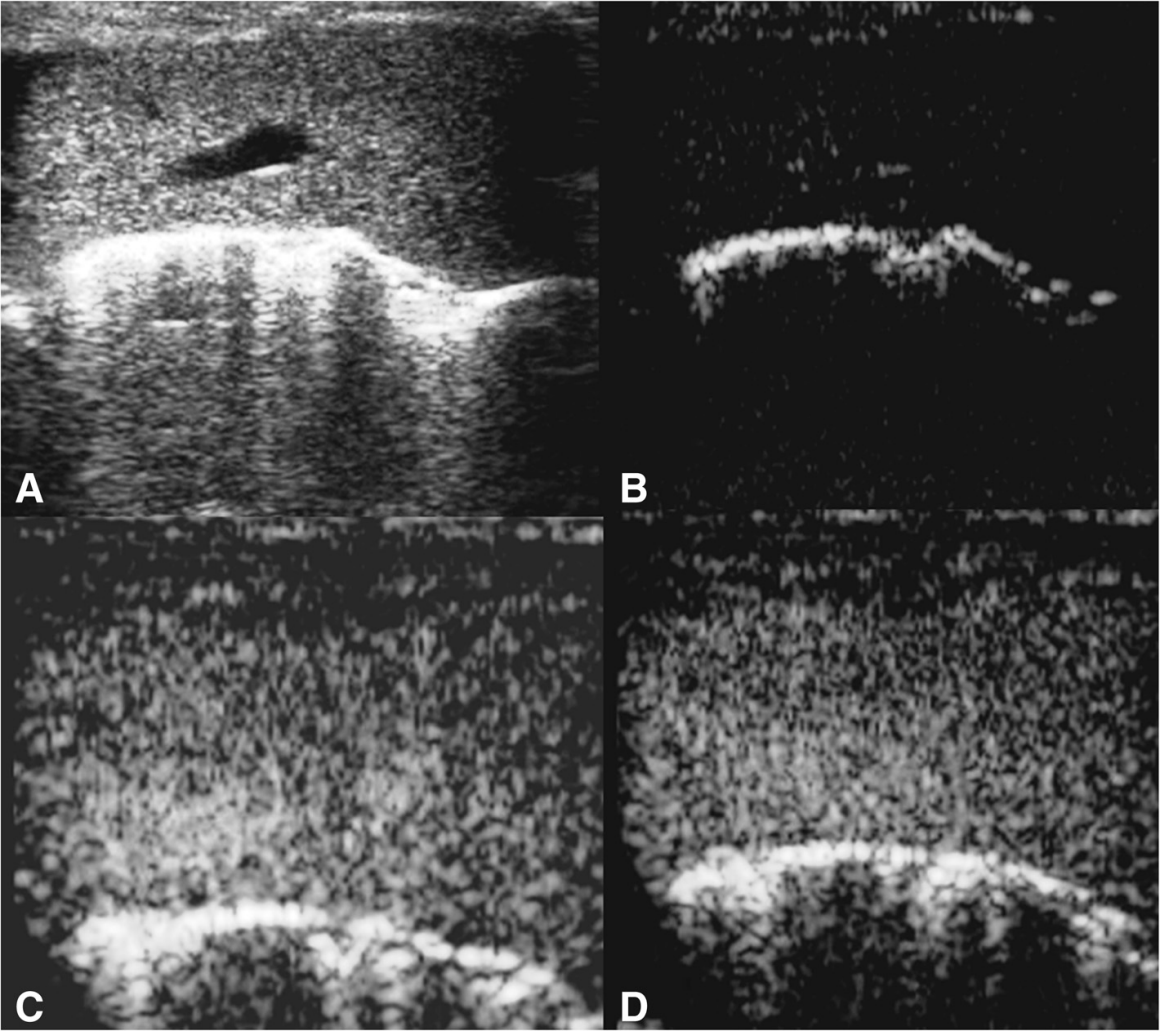
**Representative ultrasonographic evaluation of the liver of an iguana included in the study.** Artifacts secondary to the scales are visible during B-mode examination **(A)**. At T0, before injection of contrast media, **(B)** no contrast enhancement of the liver vessels is present, while enhancement it is well visible 36 **(C)** and 90 **(D)** seconds after contrast medium injection.

**Figure 2:**
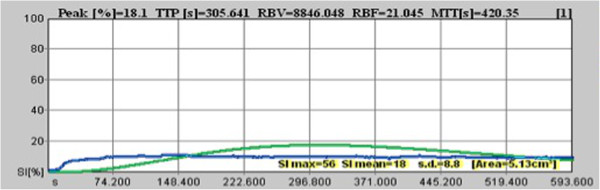
Time-intensity curve of the distribution of SonoVue in the liver of a representative green iguana.

Upon B-mode ultrasonography, the liver was easily visualized in all iguanas (Figure 1A). After the injection of contrast medium (wash in phase, Figure 1B) an increasing echogenicity in the hepatic arteries was initially observed, followed by a more diffuse and homogenous enhancement of the liver parenchyma during the portal phase, until the peak intensity was reached (Figure 1C). During the wash out phase, a homogenous decrease of the echogenicity was visible in all cases (Figure 1D). Complete clearance of the contrast medium was not achieved neither in the 3 individuals in which image acquisition ran for 10 minutes. Representative frames of the scanning sequence after contrast medium administration from one of the iguanas are shown in Figure 1, and a representative time–intensity curve over the region of interest is shown in Figure 2.

The mean peak enhancement was 19.9% ± 7.5 (18.3; 11.7-34.6). TTP_ceus_ was 134.0 ± 125.1 (68.4; 59.6-364.5) seconds. Distribution of both peak enhancement and TTP_ceus_ were normal and no outliers were detected.

Repeatability of CEUS was adequate, with intra-individual variations of peak enhancement and TTP_ceus_ of 10.5% and 1.2%, respectively.

### CECT

**Figure 3:**
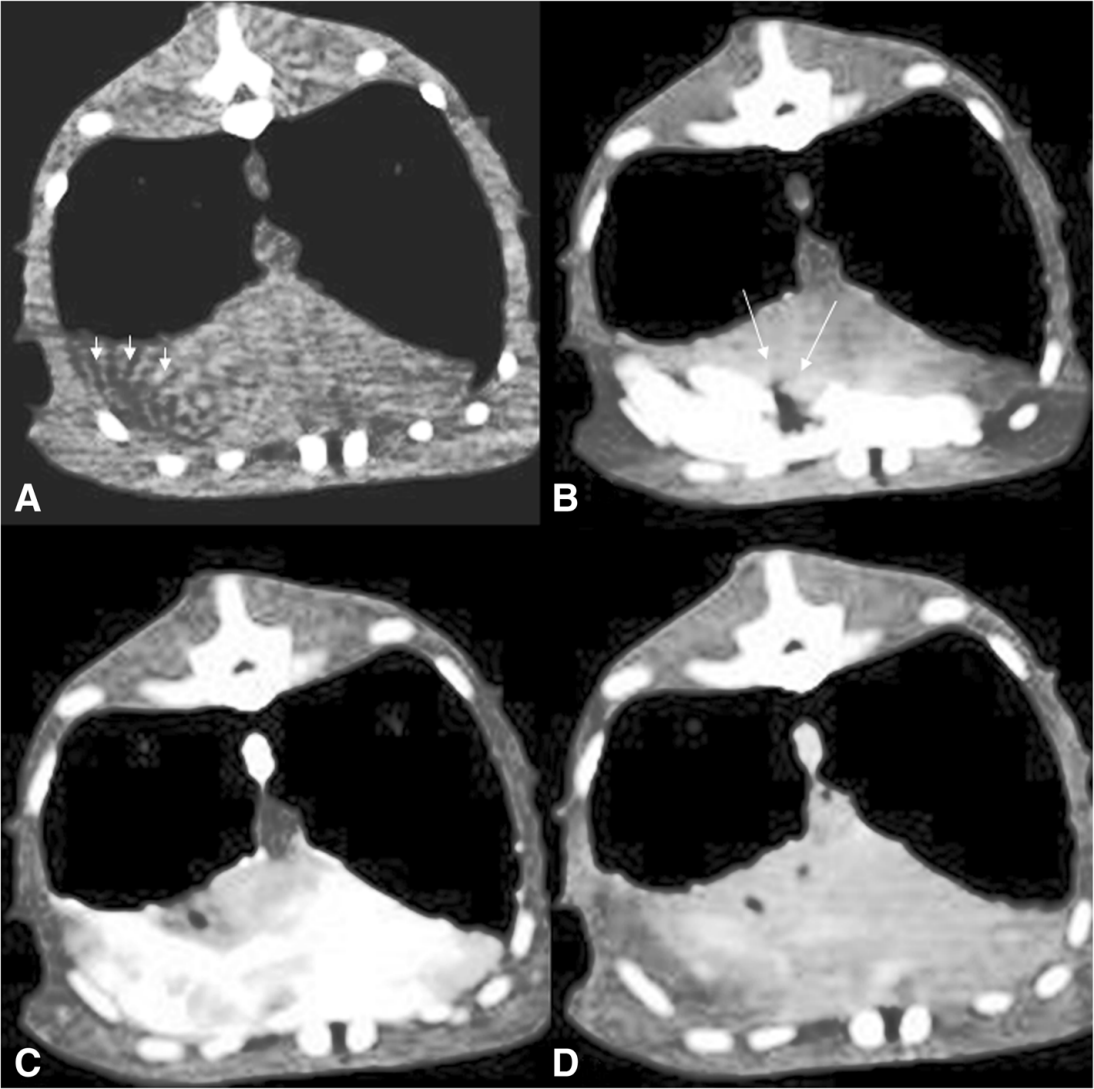
**Representative dynamic CT study of an iguana included in the study.** No vascular enhancement is present in the vessels just after the contrast medium injection **(A)**; the contrast medium is present in the caudal vena cava, but not in the aorta 3 seconds after injection **(B)**; 36 seconds after injection is well visible in all the liver parenchyma **(C)**, and after 600 seconds from the injection start, the contrast medium it is still mildly appreciable **(D)**. Beam hardening (arrowheads - **A**) and blooming (arrows – **B**) artifacts are present due to ribs, and the pool of contrast medium in the caudal vena cava respectively.

**Figure 4:**
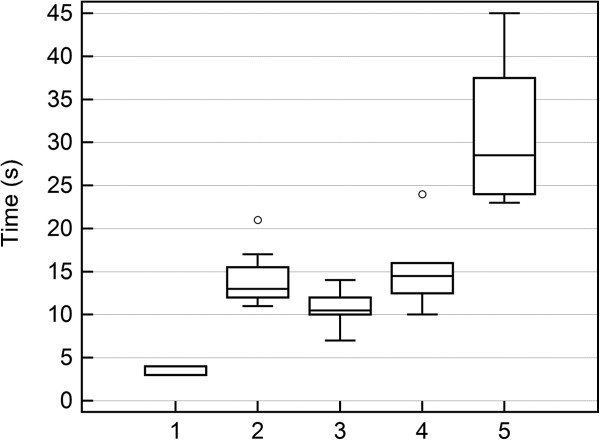
**Box-and-whisker plots of distribution of contrast medium (CM) in liver of healthy green iguana as evaluated by means of dynamic CT scan.** Appearance of CM in aorta (1), time to peak in aorta (2), appearance of CM in liver vessels (3), detection of CM in the liver (4), time to peak in the liver (5). The boxes represent the values from the first to the third quartile. The horizontal line in each box represents the median. The whiskers include values of 1.5 times the interquartile range. Dots represent values that are larger than the upper quartile plus 3 times the interquartile range.

**Figure 5:**
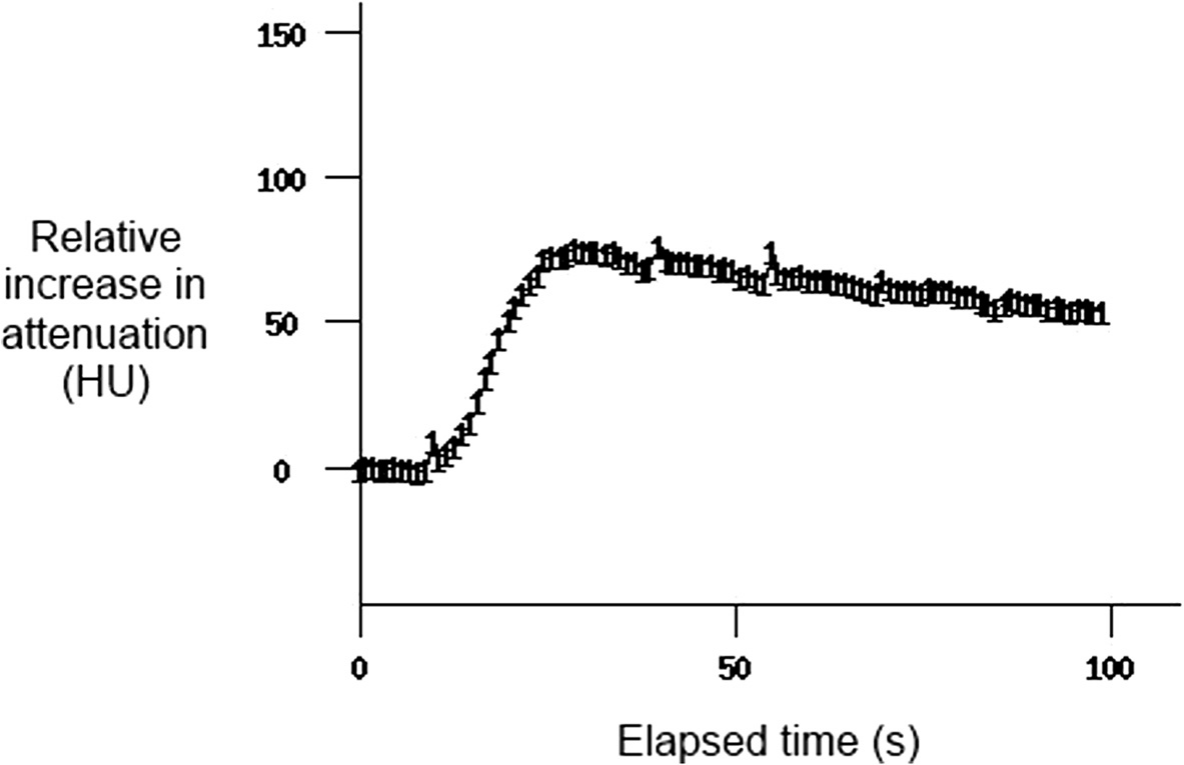
Time-density curves for aorta and liver during 600 seconds of investigation in a representative iguana.

Liver parenchyma and margins were easily visualized in the plain CT scan (Figure 3). Basal liver attenuation as measured through CT scan analysis was 77.3 ± 6.2 HU reaching 179.1 ± 35 HU at peak enhancement. The first visualization of the contrast medium was at 3.6 ± 0.5 (4; 3-4) seconds in the aorta, 10.7 ± 2.2 (10.5; 7-14) seconds in the hepatic arteries, and 15 ± 4.5 (14.5; 10-24) seconds in the liver parenchyma. TTP_cect_ in the aorta was 14.1 ± 3.4 (13; 11-21) seconds. TTP_cect_ in the liver parenchyma was 31 ± 9.6 (29; 23-45) seconds. One iguana (No. 8) was found to be an outlier, presenting slower enhancement in aorta (21 seconds) and slower arrival of contrast medium in the liver (24 seconds) than the population studied (Figure 4) and was excluded from the descriptive statistics. A time-intensity curve representative of the population studied is reported in Figure 5.

## Discussion

Results of the present study indicate that imaging techniques employing intravenous distribution of contrast media may be used in green iguanas to evaluate liver texture and perfusion. The distribution of the contrast agent within the liver either during contrast-enhanced ultrasound and contrast-enhanced computed tomography was accompanied by a clearly recognizable change in liver echogenicity and liver attenuation, respectively. More invasive techniques, such as endoscopic biopsy [13], have been previously employed to evaluate the liver in green iguanas, but to the authors knowledge, this is the first time that enhancement of liver tissue in iguanas after administration of contrast media during CT and ultrasound examinations is evaluated. Although liver biopsy is considered to be the gold standard for diagnosing hepatic diseases, it may be useful to have reference ranges for diagnostic imaging techniques that permit evaluation of liver hemodynamics.

By use of the same contrast medium employed in the present study, during CEUS examination of the liver in dogs [15,36] a (1) hepatic arterial phase at 10-25 s post-injection has been defined, followed by a (2) portal vein phase at 20-90 seconds post-injection. This last phase lasted 150-240 seconds. Such phases reflect the double vascularization of the hepatic tissue by the hepatic artery (20% to 30% of blood) and the portal vein (70% to 80% of blood) [15,37]. In green iguanas we observed a similar pattern, characterized by a (1) increased echogenicity of the hepatic arteries and (2) diffuse enhancement of the liver parenchyma, until the peak intensity was reached. The wash out phase in the iguanas was characterized by a gradual decrease in echogenicity of the liver parenchyma. In all cases enhancement of the liver lasted for more than 10 minutes.

There are two findings of the present study that are worthy of mention, as they strongly differed from what is expected in mammals. Firstly, time to peak, a measure of the time needed to the contrast medium to provide the maximum enhancement of a target organ (ie, the liver), was determined to be 134 (range 59-364) seconds in our study, drastically longer than what is described in dogs and cats. In conscious and anesthetized dogs, time to peak enhancement occurred among 15 to 46 seconds after injection of the contrast media [15,36]. In cats, time to peak occurred approximately 10 seconds after injection of the contrast agent, with a significant enhancement of liver parenchyma from approximately 10 dB of baseline intensity to approximately 30 dB of peak intensity [38]. This finding is especially relevant because (1) if results of CEUS examination of green iguanas are interpreted extrapolating current knowledge on dogs and cats, healthy green iguanas would be suspected to suffer a delay in reach of time to peak enhancement. Furthermore, (2) when performing contrast-enhanced imaging techniques to evaluate the liver in lizards, longer studies should be planned than that performed in mammals.

The second unexpected finding was the inter-individual variance observed in time to peak enhancement. In previous studies on dogs and cats, time to peak was characterized by a relatively small standard deviation: in 11 dogs the standard deviation was of 20 seconds (mean 34.6 seconds) and in 10 cats the standard deviations was of 2.9 seconds (mean 9,6 seconds) [36,38]. This suggests that time to peak is a parameter somehow consistent among individuals of the same species. In contrast, in the iguanas studied here TTP_ceus_ varied greatly among individuals with a much higher standard deviation (over 100 seconds). Due to the design of our study it is impossible to objectively determine which factor was responsible for such high inter-individual variability. We suppose that the greater variance for time to peak observed in this study may be secondary to intrinsic factors (eg, vitellogenesis, large variation in the age of the iguanas), or to the use of chemical restraint. In fact, although all the iguanas underwent the same anesthetic protocol, effect of anesthetic agents has historically been considered to show much greater variability among reptiles that in mammals [39].

In the present study all the examinations have been performed in iguanas under general anesthesia. Although in aggressive iguanas chemical restraint may be necessary to perform CEUS safely, in most occasions manual restraint should be sufficient to perform a standard CEUS examination of the liver in clinical setting. To facilitate the examination the stimulation of the vaso-vagal response, via light digital pressure applied to the eyelids, could be performed [40]. Nevertheless, it should be taken in account that results obtained in the present study may not apply to conscious iguanas or iguanas in which the baroreceptor reflex has been stimulated: In a previous study evaluating CEUS of the liver in dogs, time to peak enhancement was significantly shorter when dogs were anesthetized with propofol [36]. Such difference was suspected to be secondary to the effect that propofol has on the vascular system (ie, increase in hepatic arterial blood flow and decrease of systemic arterial pressure [41]). In any case, the other parameters did not differ in conscious and anesthetized individuals. Therefore, when considering the values produced in our study, the use of anesthetic drugs has to be considered, especially concerning time to peak enhancement.

Unenhanced CT scans provided excellent visualization of lizard’s liver. Contrast-enhanced computed tomography permitted further investigation of perfusion. Time-density curves obtained in iguanas were not dissimilar to curves that are usually obtained in mammals [42]. The graphic obtained for the aorta was characterized by a very pendant curve during wash-in with a high peak, and an initial rapid decrease followed by a slower second phase during washout. In the liver the wash-in phase was slower and the peak lower, whereas the pattern after peak was similar to that of the aorta, both of which did not complete washout during the 600 seconds of investigation. The curve pattern appeared quite similar in all animals studied apart from a few differences noted in the graphs for iguana No. 8. The distribution of the contrast medium in Iguana No. 8 was characterized by slower TTP_cect_ in aorta and slower visualization of contrast medium in the liver, resulting in an evident right shifting of the time-density curve. In all the individuals studied the contrast medium was still partially visible in the aorta and in the liver after a 600 seconds period of investigation. Such slower wash out phase compared to mammals [36] is probably caused by the lower metabolic rates of reptiles, which averages 25% to 35% of that of mammals [43].

Minimal fluctuations visualized in some of the time-intensity curves were probably associated with the respiratory acts of the iguanas. Although difficulties on keeping the ROI in the middle of the scanned area of the liver is reported also in anesthetized cats [38], we suspect that in reptiles, the fluctuations secondary to the relation between lung size and pressure in visceral organs may be more relevant due the absence of a muscular diaphragm.

In the present study absolute values for mean gray level were reported only when strictly necessary, as these values may be affected by several variables such as gain setting, mechanical index, scanning depth, the size and body composition of the individual animal, and the behavior of the individual contrast medium [36]. Changes in attenuation of the liver are for example described in chelonians, in which hypoattenuating liver (ie, < 20 Hounsfield Units) was associated with hepatic lipidosis [44]. Considering that a moderate grade of liver steatosis was present in the individuals studied, it is possible to hypothesize that iguanas without any degree of steatosis may present a more attenuating liver parenchyma.

Another factor that should be taken in account, whenever the present study is used as a reference, is that the contrast medium was injected into the ventral tail vein. The other reasonable vascular accesses in iguanas are the cephalic vein and the jugular vein, although they usually require a surgical incision of the skin [45]. Some differences in time to peak enhancement may be expected if injection of the contrast medium is performed in a different vein due to the different endovascular transit done by the contrast medium.

Proper cohort or case-control studies in iguanas would be ideal to identify an increased risk in mortality or in adverse events in animals undergoing CEUS or CECT [46]. Such typology of studies is rarely performed in reptile medicine due to the overall limited number of reptile patients, and to the multitude of confounding factors that should be considered (eg, species, metabolism, housing). Nevertheless, based on the lack of complications in the present study and based on evidence recently acquired in dogs and cats [47], there is no indication to suppose that these techniques are harmful.

## Conclusions

Normal liver attenuation and perfusion were determined by evaluation of the contrast medium diffusion in the parenchyma. Time-density curves of the liver were characterized by a fast time to peak and a slow wash out which was not completed during the recorded time. An important inter-individual variation is present in clinically healthy iguanas. However, due to the small population sampled in this study we cannot determine whether this is a consistent phenomenon in this species.

Further studies in iguanas with hepatic diseases are needed in order to evaluate potential differences in the attenuation between normal and abnormal livers. In particular, inclusion of lizards with focal hepatic lesions would be aimed, as characterization of focal lesions is one of the most powerful applications of contrast-enhanced imaging techniques. Lastly, future studies including captive and wild iguanas may allow evaluation of whether the moderate lipidosis observed in the hepatic samples was secondary to housing or dietary conditions.

## Methods

### Animals

Clinically healthy, client-owned, captive-born green iguanas (*Iguana iguana*) presented to the Clinica Veterinaria Modena Sud (Spilamberto, MO, Italy) in a three-month period were eligible for inclusion in the study. To be included in the study iguanas needed to have no previous pathologies reported and to be maintained in proper dietary (ie, fed mixtures of leafy green vegetables and had access to water ad libitum) and housing conditions (ie, presence of a basking spot reaching 35°C [95°F], UVB lamps replaced at least biannually, exposure to natural sunlight in spring and summer). The animals were considered healthy on the basis of physical examination and of clinical biochemistry values within published reference ranges [48,49]. Biochemistry was performed by means of a bench-top analyzer (VetScan, Abaxis, Inc., Union City, CA) that use commercially available rotor designed to be used in Avian/Reptilian patients. Parameters analysed for each animal were albumin, aspartate transaminase, biliary acid, calcium, creatine kinase, glucose, phosphorus, potassium, sodium, total protein, and uric acid.

The study was performed in compliance with the directive 2010/63/EU of the European parliament and of the European council. The institutional ethical committee of the University of Bologna approved all the procedures. The owners gave written informed consent for the enrolment of their animals in the study.

### Procedures

The ventral tail vein of each iguana was catheterized using a 22 gauge IV catheter (Jelco, Smiths Medical International Ltd, Lancashire, UK) inserted at two-thirds of the tail length. Anesthesia was induced with a slow intravenous 10 mg/kg [4.54 mg/lb] injection of propofol (Fresenius Kabi, Isola della Scala, Italy) [50] with the animals maintained in a warm room (28°C [82.4°F]) for 24 hours before the procedure. Iguanas were intubated with non-cuffed tracheal tube (with diameter between 2.0 and 3.0 mm) and connected to a closed Y pediatric circuit. Anesthesia was maintained administering 2.0% isoflurane and 0.8-1.2 litre/minute oxygen through an adjustable dial (concentration range, 0-5%) coupled with a separate oxygen flow meter (range, 0.2-4 L/min). Manual ventilation was performed if apnea lasted more than 20 seconds. The heart rate was monitored through a doppler probe placed on the jugular vein during the entire procedure. Ultrasound and CT scans were performed as described below. Following the imaging session ultrasound-guided liver biopsies were obtained from each iguana. After the procedures the iguanas were individually placed in a small warm enclosure (32°C [89.6°F]) and closely monitored during recovery from anesthesia.

### Ultrasonography procedures

A survey liver scan was performed using standard B-mode ultrasonography with 5-7.5 MHz linear transducer with coded harmonic capability (Esaote Mylab 30, Esaote-CnTI System, Esaote, Genova, Italy), to ensure there were no visible liver lesions, and to permit selection of a suitable acoustic window, i.e., one that provided an uninterrupted view of as large a section of liver parenchyma as possible. The animals were maintained in ventro-dorsal position, and large amount of gel was used in order to reduce artifacts caused by entrapment of air bubbles in between the scales.

A second-generation contrast agent composed of sulphur hexafluoride microbubbles (SonoVue 8 mcl/ml, Bracco Imaging S.p.A., Milan, Italy) and a dedicated contrast-enhanced ultrasound analytical software (Contrast Tuned Imaging, Contrast Tuned Imaging technology, Esaote, Genova, Italy) were used. When an appropriate acoustic window was found, a rapid bolus dose of 0.03 ml/kg of the contrast medium was injected through the IV catheter followed by a rapid bolus of 1.5 ml of saline (0.9% NaCl). The timer was activated at the moment of the injection (T = 0) and the flow of contrast into the liver was observed in real-time and digitally recorded for 1 minute and 30 seconds. On the first 5 iguanas two consecutive CEUS examinations were performed 30 minutes apart to assess repeatability of the method. As results of the first 5 examinations showed that in 1.5 minutes there was no clearance of the contrast medium in the liver, recording was prolonged to 10 minutes.

Videos were analyzed using specific software (Qontrast, Esaote, Italy) to generate time-intensity curves for each exam. The peak enhancement and time to peak (TTP_ceus_) were calculated for each individual. Perfusion parameters were defined as follows: peak enhancement (maximum signal intensity reached during the transit of the bolus, expressed in % where 100% means the maximum), TTP_ceus_ (time of arrival of contrast agent post-injection to its maximum peak enhancement value).

### CT procedures

Dynamic CT images were obtained by use of a multidetector 16 slices CT scanner (BrightSpeed 16, GE Corporate, Milwaukee, WI) with the animals being in ventral recumbence. After the plain images were taken, a section of the liver close to the hilum was chosen and one image was taken every second starting from the injection time for a total of 600 images, in order to obtain curves of liver perfusion enhancement. A dose of 800 mg/kg of an iodinated contrast medium (Ioversol 320 mg/ml, Optiray, Covidien Spa, Italy) [42], was injected trough the ventral tail vein, at 3 ml/sec, using a power injector (Optistar injector, Mallinckrodt plc, Dublin, Ireland).

Liver attenuations in the plain studies and at peak enhancement were determined. Time of first visualization of contrast media in the aorta, in the hepatic arteries, and in the liver parenchyma was recorded. Times to peak (TTP_cect_) in aorta and in the liver parenchyma were also determined by visual inspection of the curves.

### Histopathological procedures

A biopsy device (Spirotome 10 G, Medinvents NV, Hasselt, Belgium) composed by cutting cannula, trocar, helical tissue receiving needle, and releasing device with a cut length of 18 mm was used to obtain liver biopsies in the iguanas, according to the technique previously described in companion animals [51]. A small cutaneous incision was performed with a number 11 scalpel blade in the iguanas to facilitate the introduction of the device through the skin. The biopsies were taken both from the right and the left lobe, distant from any visible vessel. A total of 16 biopsies (two per each animal), were collected by a board-certified radiologist (MV).

Tissue samples were immediately placed into 2 ml screw-top plastic tubes containing 10% buffered formalin. The tissues were then routinely processed and stained with hematoxylin and eosin. Tissue sections were examined by a board-certified veterinary pathologist (FCO).

### Statistical analysis

Statistical analysis was performed by use of a commercial software (MedCalc 12.2.1, MedCalc Software, Mariakerke, Belgium). Data are reported as mean ± SD (median; range) unless otherwise stated. P-values less than 0.05 were considered significant. Non-normality was investigated for each parameter through the D’Agostino-Person test. Tukey method was employed to detect outliers, i.e., values smaller than the lower quartile minus 1.5 times the interquartile range, or larger than the upper quartile plus 1.5 times the interquartile range. Repeatability of TTP_ceus_ and CEUS peak enhancement was measured calculating the CV from duplicate measurement [52].

## Competing interests

The authors disclose any financial interests with companies that manufacture products that are the subject of the present research or with companies that manufacture competing products.

## Authors’ contribution

GN, AZ and MV conceived the study. GN cared for the iguanas, performed the venous catheterisation, analysed the images and assisted in drafting the manuscript. ND analyzed the data and drafted the manuscript. SL assisted with the anesthesia of the iguanas and wrote a first draft of the manuscript. IP assisted with the ultrasonographic procedures. FCO analyzed the liver biopsies. MV performed the diagnostic examinations, performed the liver biopsies, analyzed the images and assisted in drafting the manuscript. All authors read and approved the final manuscript.
